# Effects of Extract from Solid-State Fermented *Cordyceps sinensis* on Type 2 Diabetes Mellitus

**DOI:** 10.1155/2012/743107

**Published:** 2012-02-29

**Authors:** Wei-Chih Kan, Hsien-Yi Wang, Chih-Chiang Chien, Shun-Lai Li, Yu-Chun Chen, Liang-Hao Chang, Chia-Hui Cheng, Wan-Chen Tsai, Jyh-Chang Hwang, Shih-Bin Su, Li-Hsueh Huang, Jiunn-Jye Chuu

**Affiliations:** ^1^Division of Nephrology, Department of Medicine, Chi-Mei Medical Center, Tainan 71004, Taiwan; ^2^Medical Laboratory Science and Biotechnology, Chung Hwa University of Medical Technology, Tainan 71703, Taiwan; ^3^Department of Sport Management, College of Leisure and Recreation Management, Chia Nan University of Pharmacy and Science, Tainan 71710, Taiwan; ^4^Department of Food Nutrition, Chung Hwa University of Medical Technology, Tainan 71703, Taiwan; ^5^Institute of Biotechnology, Southern Taiwan University, Tainan 71005, Taiwan; ^6^Institute of Biomedical Engineering, Southern Taiwan University, Tainan 71005, Taiwan; ^7^Department of Family Medicine, Chi-Mei Medical Center, Tainan 71004, Taiwan; ^8^Department of Nephrology, Sin-Lau Hospital, Tainan 70142, Taiwan; ^9^Department of Pharmacy, Wei Gong Memorial Hospital, Miaoli 35159, Taiwan

## Abstract

Diabetes mellitus is the most common chronic disease in the world, and a wide range of drugs, including Chinese herbs, have been evaluated for the treatment of associated metabolic disorders. This study investigated the potential hypoglycemic and renoprotective effects of an extract from the solid-state fermented mycelium of *Cordyceps sinensis* (CS). We employed the KK/HIJ diabetic mouse model, in which the mice were provided with a high-fat diet for 8 weeks to induce hyperglycemia, followed by the administration of CS or rosiglitazone for 4 consecutive weeks. Several parameters were evaluated, including changes in body weight, plasma lipid profiles, oral glucose tolerance tests, insulin tolerance tests, and plasma insulin concentrations. Our results show that the CS extract significantly elevated HDL/LDL ratios at 4 weeks and decreased body weight gain at 8 weeks. Interestingly, CS treatment did not lead to obvious improvements in hyperglycemia or resistance to insulin, while in vitro MTT assays indicated that CS protects pancreatic beta cells against the toxic effects of STZ. CS also enhanced renal NKA activity and reduced the accumulation of mesangial matrix and collagen deposition. In conclusion, CS extract can potentially preserve **β**-cell function and offer renoprotection, which may afford a promising therapy for DM.

## 1. Introduction

Diabetes mellitus (DM) is the most common chronic disease in the world, and the number of diabetic patients is rapidly increasing each year. Diabetes-related complications impose a huge healthcare burden [1, 2]. Approximately 90% of diabetic patients have type 2 DM, in which insulin resistance plays a central role. Type 2 DM can induce insulin resistance syndrome, which is associated with obesity, hyperlipidemia, hypertension, atherosclerotic heart disease, and other systemic diseases [3, 4]. Developing new therapeutic strategies for hyperglycemia and insulin resistance in these diabetic patients is thus an important issue in public health worldwide.

Animal models play a major role in research related to diabetes. Appropriate animal models are essential tools for the study of type 2 DM pathogenesis, its complications, the genetic and environmental influences that increase its risk, and the development of therapeutic agents [5–7]. Several types of gene knock-out mice have been developed as animal models for type 2 DM in recent years [8–10]. The KK/HIJ mouse model is a well-established animal model for diabetes. In this study, we fed KK/HIJ mice a high-fat diet (HFD) for 8 weeks to induce hyperglycemia. We then studied the effects of extracts from *Cordyceps sinensis* in this KK/HIJ mouse model of diabetes.

*Cordyceps sinensis* (CS), also called Dong Chong Xia Cao (winter worm summer grass) in Chinese, is one of the most valued herbs in traditional Chinese medicine [11–15]. In previous studies, CS has been proven to have significant hypoglycemic activity in genetic and streptozotocin-induced diabetic mice and rats [16, 17]. Recent reports also indicate that the fermented mycelia of CS have anti-hyperglycemic activities similar to those of CS fruiting bodies in rats with induced type 1 diabetes [18].

Diabetic nephropathy (DN) is one of the major forms of chronic kidney disease (CKD). It is a common and serious complication of diabetes mellitus, leading to renal failure in up to 30% of individuals with diabetes; DN has thus become one of the most important end-stage renal diseases (ESRD). A nongenetic model of type 2 DM has been described that involves feeding with a high fat diet and administering a low dose of STZ, resulting in insulin-resistance and hyperlipidemia in mice and rats [14]. One clinical study has demonstrated that renal function in patients with chronic allograft nephropathy (CAN) significantly improved after 6 months of treatment with CS [19]. One in vitro study also indicated that CS can reduce angiotension-2-induced renal tubular epithelial cell apoptosis [20].

Despite these reports, there is still little information regarding the effects of CS on the pancreas and kidneys in animal models of type 2 DM. We therefore designed this in vivo pharmacological study to determine the effects of CS on blood glucose, cholesterol triglycerides, HDL/LDL, glucose tolerance, and insulin tolerance in diabetic KK/HIJ mice. The aim of this study was to investigate the potential effects of fermented CS mycelium extract on diabetes, including pancreatic *β*-cell preservation and protection of kidney function.

## 2. Materials and Methods

### 2.1. Chemicals and Reagents

Culture medium RPMI-1640, fetal bovine serum, sodium bicarbonate, L-glutamine, and 0.05% trypsin-EDTA were purchased from Life Technologies (Carlsbad, CA., USA). Streptozotocin (STZ), sulforhodamine B sodium salt (SRB), and rosiglitazone (RS) were from Sigma (Saint Louis, Missouri, USA). Mouse monoclonal antibodies-PPAR*γ* and HRP anti-mouse IgG were from Santa Cruz Biotechnology, Inc (Santa Cruz, CA, USA). Nitrocellulose membranes were purchased from NEN Life Science Products (Boston, MA, USA). Mouse insulin and glucose ELISA kits were from Mercodia (Sylveniusgatan, Uppsala, Sweden).

### 2.2. Preparation of *Cordyceps sinensis* Crude Extract

Commercial pulverized crude extract from the solid-state fermented mycelium of *Cordyceps sinensis *(CS) used in this study was provided by Blue Ocean Universal Bio-Tec Co., Ltd. (Taipei, Taiwan). The CS was extracted with 95% alcohol by stirring overnight at room temperature; after filtering, the residues were extracted twice with 95% alcohol using the above procedure. The CS extract was then concentrated and dehydrated by freeze-drying (Kingmech, FD 20L-6S, Taiwan). The extraction rate was 25.6%. The solid-state (solid) fermented CS extract contained no fruiting bodies, and a dose of 300 mg/kg/day equals approximately 300 mg/kg/day of CS mycelia.

### 2.3. Animal Preparation

Six-week-old male KK/HIJ mice were purchased from the Jackson Laboratory (Biolasco, Taiwan). All animals were maintained in laminar flow cabinets under specific pathogen-free conditions in facilities approved by the Accreditation of Laboratory Animal Care and in accordance with the Institutional Animal Care and Use Committee (IACUC) of the Animal Research Committee in the Chi-Mei Medical Center, Tainan, Taiwan. The cages, bedding, food, and water were autoclaved. Animals were maintained on a daily 12 hr light/12 hr dark cycle. The KK/HIJ mouse is an obese diabetic model in which the Ay mutation is introduced into a KK strain background. To simulate type 2 DM, KK/HIJ mice were fed a high-fat diet (HFD) consisting of 40% (wt/wt) fat for 8 consecutive weeks, while other KK/HIJ mice were given normal chow as a control. CS (300 mg/kg/day) and RS (0.5 mg/kg/day) were administered orally to diabetic KK/HIJ mice for 2 or 4 consecutive weeks.

During experiments, the body weight was recorded regularly and blood samples were drawn from the retro-orbital sinus of animals weekly for monitoring of the nonfasting plasma glucose levels (2 hr postprandial hyperglycemia). All enrolled mice, in groups of 16, were weighed at 0, 2, and 4 weeks. After sacrificing 8 mice per group for examination, the body weight of the remaining 8 mice was recorded at 8 and 12 weeks.

### 2.4. Determination of Biochemical Indexes

After 2 or 4 weeks of being fed a high-fat diet, treatments with CS and RS were begun in diabetic KK/HIJ mice. Blood samples were prepared and centrifuged, and blood glucose levels were analyzed using MAJOR II (Taipei, Taiwan); insulin was measured using an insulin ELISA kit (Mercodia AB, Sweden). Serum levels of total plasma cholesterol (TC), high-density-lipoprotein cholesterol (HDL-C), low-density-lipoprotein cholesterol (LDL-C), and triglycerides (TG) were analyzed using a blood chemistry analyzer (Hitachi 7040).

### 2.5. Oral Glucose Tolerance Test (OGTT)

After an overnight fast, 3 g/kg D-glucose was administered orally to KK/HIJ mice (normal diet), and the mice (HFD) treated daily with CS, RS, and control, respectively, for 4 consecutive weeks. To measure the fasting blood glucose levels, the blood samples were collected from each subject at 0, 15, 45, 95, and 135 minutes relative to the start of the oral glucose administration. The KK/HIJ mice (normal diet) served as native controls in comparison to the KK/HIJ mice (HFD), treated with CS, RS, or saline.

### 2.6. Insulin Tolerance Test

After fasting overnight, 0.5 U insulin was administered intraperitoneally to all mice daily for 4 consecutive weeks. To measure fasting blood glucose levels, blood samples were collected from each subject at 0, 15, 45, 95, and 135 minutes relative to the start of the oral glucose administration. The KK/HIJ mice (normal diet) served as native controls in comparison to the KK/HIJ mice (HFD).

### 2.7. In Vitro Cell Culture

Rat insulinoma cells (RIN-m5F) were purchased from the American Type Culture Collection and maintained in RPMI-1640 medium supplemented with 10% fetal bovine serum, 2 mM L-glutamine, 1.5 g/L sodium bicarbonate, 4.5 g/L glucose, 10 mM HEPES, and 1.0 mM sodium pyruvate. For experiments, cells were subcultured into T75 Flask Equivalents; the medium was changed every 2 days.

### 2.8. Cell Toxicity Assessment

The viability of rat pancreatic insulinoma cells (RIN-m5F) was measured by the reduction of MTT [3-(4,5-dimethylthiazol-2-yle) 2,5-mdiphenyl-tetralozium bromide]. Cells were harvested when in the exponential growth phase, then cultured in 100 *μ*L of fresh medium at 5 × 10^4^ cells/well in 96-well plates. Cells were incubated in 5% CO_2_ at 37°C. After 24 hours, the suspensions were removed and coincubated with CS extracts and STZ for 48 and 72 hours, respectively.

Cell proliferation was then assayed using the MTT assay as follows. PBS solution (20 *μ*L) containing 5 mg/mL MTT was added to each well; cells were further incubated for 4 hours then solubilized in 100 *μ*L DMSO for optical density measurements at 570 nm. Cell proliferation was expressed as the ratio of the number of MTT-treated cells to the number of control cells (% of control).

### 2.9. Western Blotting Analysis

At the end of the experiment, all mice were sacrificed and their livers prepared for the analysis of expression of proteins relevant to insulin resistance*.* The tissues were ground, centrifuged, and incubated in protein extraction buffer for 1 hour. Samples were centrifuged at 14 000 rpm for 30 min. Proteins were separated by 10% PAGE, then transferred onto nitrocellulose membranes. After blocking, membranes were incubated with anti-PPAR-*γ* antibody (1 : 200) for 1 hour at room temperature in rinse buffer, followed by four washes of 10 minutes each. Horseradish peroxidase-conjugated anti-rabbit IgG and anti-mouse IgG antibodies were diluted 1 : 5000 in rinse buffer and incubated with blots for 1 hour at room temperature. Horseradish peroxidase was added (Reagent A + Reagent B, 1 : 1 ratio) to visualize antibody-bound PPAR*γ* protein on the NC membrane. Western blot images were obtained using a LAS-3000 analyzer (Fuji, Japan).

### 2.10. Na^+^/K^+^-ATPase Activity Assay

Membrane ATPase activity was assayed. The method used allowed the distinct quantification of Na^+^/K^+^-ATPase and Mg^2+^-ATPase activities as a result of extracting all proteins from the renal cortex. The enzymatic activities were measured in triplicate in covered 96-well microtiter plates at  37 ± 0.5°C on a shaker. Thirty microliters of assay buffer (118 mM NaCl, 1.67 mM KCl, 1.2 mM MgCl_2_, 12.3 mM NaHCO_3_, 11 mM glucose, 0.5 mM EGTA, 3 mM ATP, and 1.25 mM ouabain, pH 7.4) was added to each well. The plates were read on an ELISA microplate reader at 630 nm. The absorbance values obtained were converted to activity values by linear regression using a standard curve for sodium monobasic phosphate that was included in the assay kit. The Na^+^/K^+^-ATPase activity was determined by subtracting the ouabain-insensitive (Mg^2+^-ATPase) activity from the overall Na^+^/K^+^/Mg^2+^-ATPase activity level. Values reported represent the mean and SE of at least three separate experiments.

### 2.11. The Masson Trichrome Staining

Kidney samples were fixed in 10% formal-saline for 48 hours, and then dehydrated by successively passing through a gradient of mixtures of ethyl alcohol and water. The samples were rinsed with xylene and embedded in paraffin. Kidney sections (5 *μ*m thick) were prepared and stained with biebrich scarlet-acid fuchsin solution for 15 minutes, then transferred directly to aniline blue solution and stained for 5–10 minutes. Finally, the sections were mounted using neutral deparaffinated xylene (DPX) medium for microscopic examination on a Motic BA 400 microscope using Motic Advance 3.0 software.

### 2.12. Statistical Analysis

Values represent mean ± SE with 8 mice per group. Differences between groups were analyzed with an analysis of variance (ANOVA) followed by one-way ANOVA and analyzed using Sigma Plot 10.0 software (SPSS Inc., Chicago, IL, USA). Each value is expressed as the means ± SE of 8 mice. *represented *P* < 0.05, significantly different compared with native control of KK/HIJ mice. ^#^represented *P* < 0.05, compared with the samples separated for 48 hours.

## 3. Results

### 3.1. The HDL/LDL Ratio, Cholesterol, Triglyceride, and Body Weight

**Figure 1:**
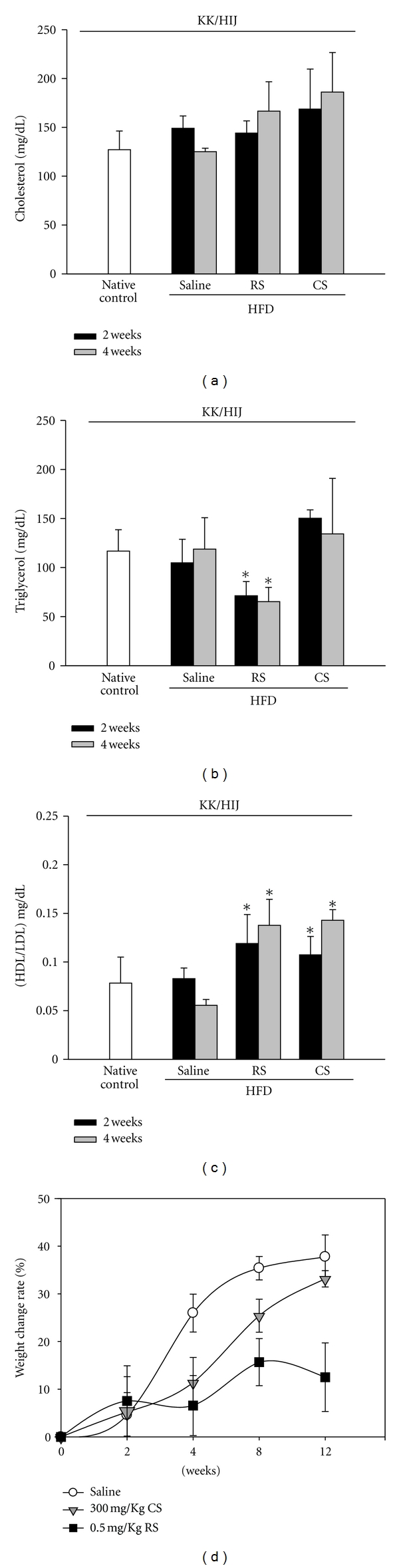
The blood biochemical index (cholesterol, triglyceride, and HDL/LDL ratio) and body weight of KK/HIJ mice. Blood samples were collected from the retroorbital sinus and the cholesterol (a), triglyceride, (b) and HDL/LDL (c) levels were measured following administration of 300 mg/kg/day of *Cordyceps sinensis* (CS), 0.5 mg/kg/day of rosiglitazone (RS), or saline (control group) for 4 consecutive weeks in HFD-treated KK/HIJ mice. Blood chemistry was analyzed on weeks 2 and 4 using a blood analyzer (Hitachi 7040). The body weight gain was recorded at 0, 2, 4, 8, and 12 weeks (d). Data are mean ± SE (*n* = 8 for each group); *P < 0.05, as compared to the control group.

Consecutive administration of 300 mg/kg/day of* Cordyceps sinensis *(CS) for 4 weeks had no obvious effect on cholesterol (Figure 1(a)) and triglyceride (Figure 1(b)) levels compared to normal controls (normal diet) or the control group (HFD), while 0.5 mg/kg/day of rosiglitazone (RS) treatment reduced triglyceride levels at 2 and 4 weeks in KK/HIJ mice. However, CS and RS treatments both significantly elevated the HDL/LDL ratio compared to the control group (HFD) at 4 weeks (Figure 1(c)). During weeks 4–8, mice were not administered CS or RS, but their body weight began to increase in the CS and control groups. The data also showed a much slower body weight gain in the RS group, with this trend continuing through the end of the 12-week observation period (Figure 1(d)).

### 3.2. Glucose Tolerance Test

**Figure 2:**
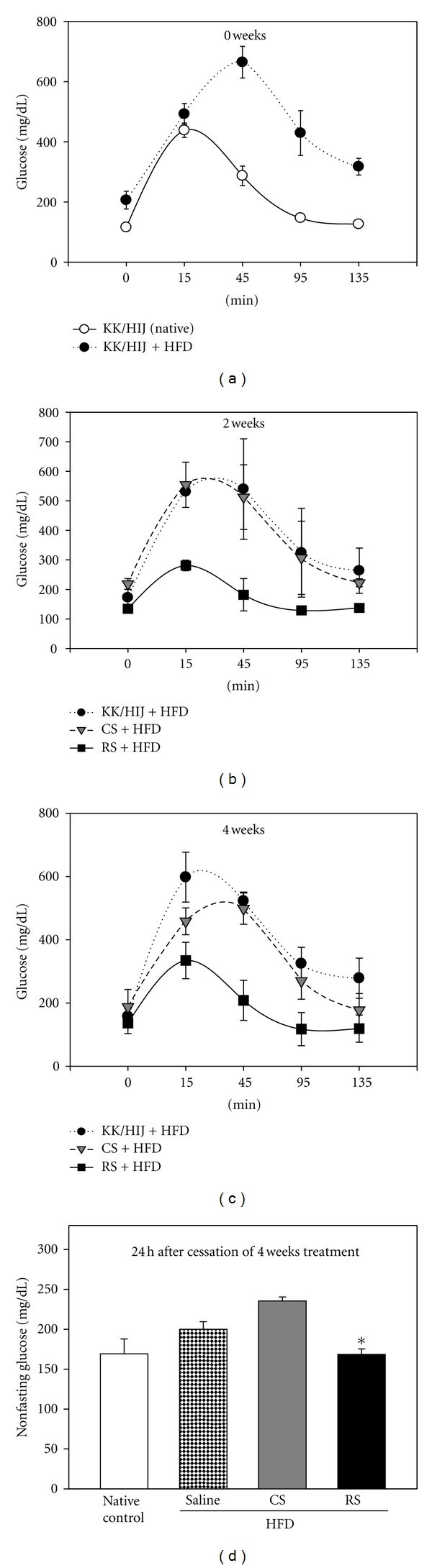
Effects of *Cordyceps sinensis* extract on oral glucose tolerance test. After 8 weeks of being fed a high-fat diet, KK/HIJ mice were treated with 300 mg/kg/day of CS extract and 0.5 mg/kg/day of rosiglitazone (RS) for 4 consecutive weeks and were compared to native controls (normal diet) and control group (HFD) after 0 (a), 2 (b), and 4 (c) weeks. After an overnight fast, 3 g/kg D-glucose was administered orally. Blood samples were obtained for evaluation of glucose levels at 0, 15, 45, 95, and 135 min. Nonfasting glucose values (2 hr postprandial hyperglycemia) (d) were also determined 4 weeks later using a glucometer on blood samples. Data are mean ± SE (*n* = 8 for each group); *P < 0.05, as compared to the control group.

Glucose intolerance in mice fed a HFD was more severe than in mice fed a normal diet (Figure 2(a)). After 2 weeks of treatment with CS or RS, sequential blood glucose levels declined only in the RS group (Figure 2(b)). For 4 consecutive weeks, mice in the RS group progressively increased in their sensitivity to glucose; meanwhile the difference in glucose tolerance between the CS and control groups was also revealed throughout the study period, being at 0, 15, 45, 95 and 135 min (Figure 2(c)). Accordingly, at 4 weeks, the nonfasting results indicate that the total glucose levels were significantly decreased by RS but not by CS (Figure 2(d)).

### 3.3. Insulin Tolerance Test

**Figure 3:**
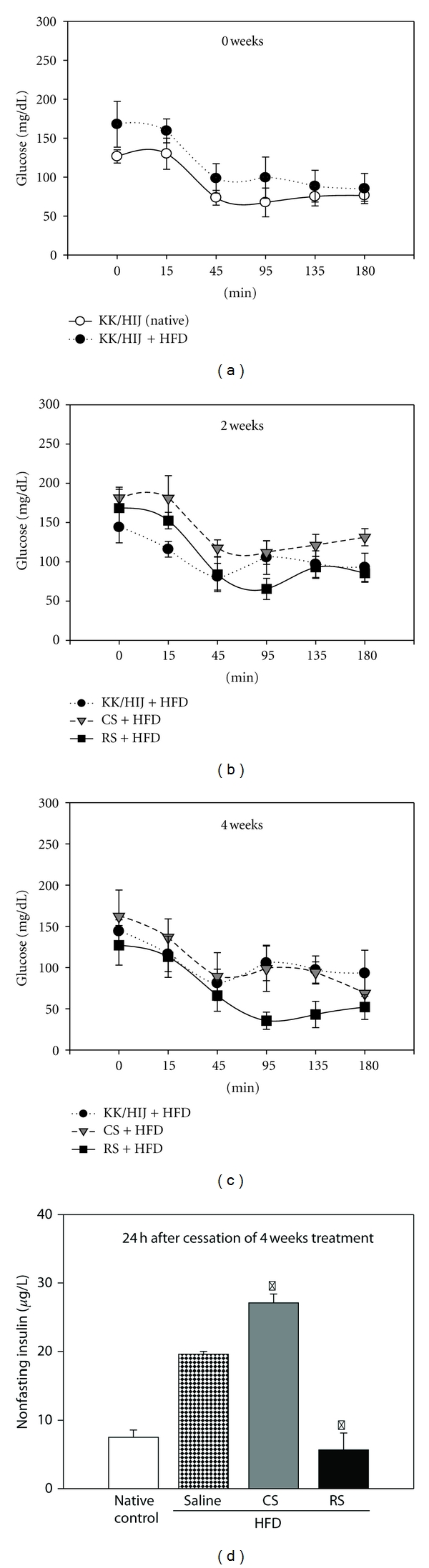
Effect of *Cordyceps sinensis* extract on insulin tolerance test (ITT). After 8 weeks of being fed a high-fat diet, KK/HIJ mice were treated with 300 mg/kg/day of CS extract and 0.5 mg/kg/day of rosiglitazone (RS) for 4 consecutive weeks and were compared to native controls (normal diet) and control group (HFD) after 0 (a), 2 (b), and 4 (c) weeks. After an overnight fast, 0.5U insulin was administered intraperitoneally. The blood samples were obtained to determine glucose concentrations at 0, 15, 45, 95, and 135 min. Nonfasting serum insulin levels (d) were determined using an insulin ELISA kit 4 weeks later. Data are expressed as mean ± SE (*n* = 8 for each group); *P < 0.05, as compared to the control group.

Figure 3(a) shows the changes in blood glucose levels after an insulin injection in native KK/HIJ mice (normal diet) and the control group (HFD) on day 0. The HFD control group showed elevations in fasting blood glucose levels over that of mice fed a normal diet at all time points tested after insulin administration. Results indicate that, after 2 weeks of treatment, neither CS nor RS significantly decreased fasting blood glucose levels (Figure 3(b)). After 4 weeks of treatment, mice treated with RS were less insulin tolerant at the later time points (45–180 minutes) compared to mice in the control and CS groups (Figure 3(c)). After 4 weeks of drug administration, plasma nonfasting insulin levels were increased in CS group mice and decreased in RS group mice compared with that of controls (Figure 3(d)).

### 3.4. CS Protection of Pancreatic Beta Cells against STZ Cytotoxicity

**Figure 4:**
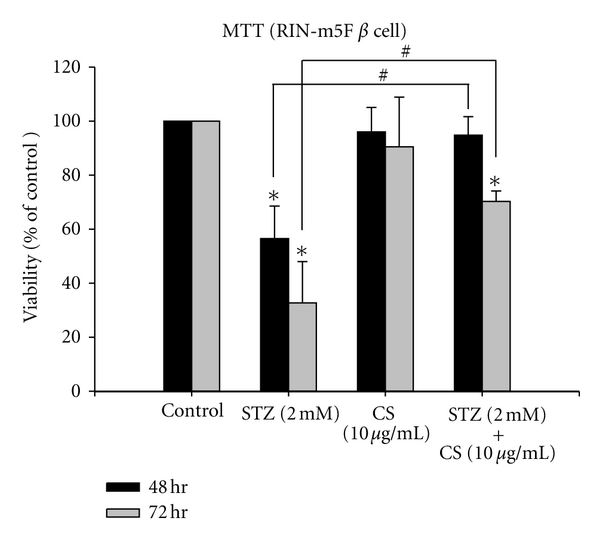
The viability of pancreatic beta cells (RIN-m5F) in vitro. The viability of beta cells (RIN-m5F) was measured after 48 or 72 hours of incubation with either STZ (2 mM), CS extract (10 *μ*g/mL), or combined combination of STZ (2 mM) with CS extract (10 *μ*g/mL). Each value represents the mean ± SE of three replicate experiments and expressed as population growth. *P < 0.05 differed from saline control. ^#^ *P* < 0.05 differed significantly from STZ treatment alone.

The viability of mouse pancreas insulinoma beta cells (RIN-m5F) was used to determine whether CS confers protection against STZ toxicity. Incubation of beta cells with STZ alone resulted in a decrease in cell viability to 56% and 32% of controls after 48 and 72 hrs of incubation, respectively (Figure 4). When co-treated with STZ and CS, beta cells demonstrated a marked increase in cell survival over those treated with STZ alone.

### 3.5. PPAR-*γ* Protein Expression

**Figure 5:**
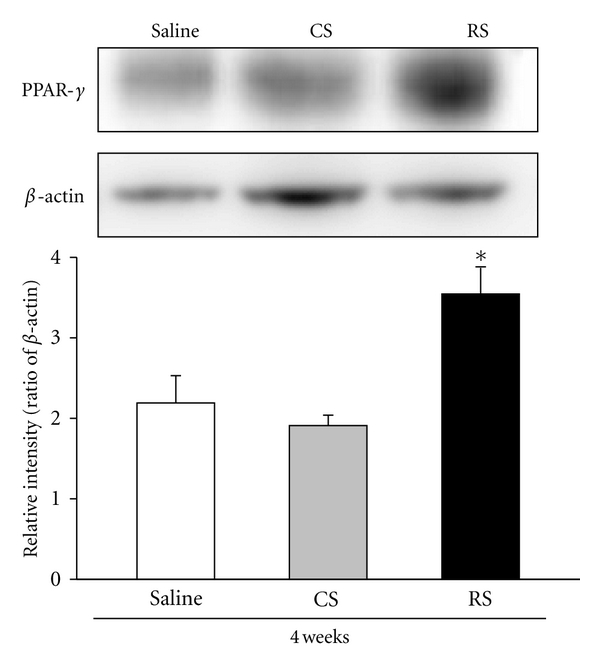
PPAR-*γ* protein expression in liver tissue. Expression levels of the PPAR-*γ* and *β*-actin proteins were determined by western blot and quantitated by microcomputer image device (MCID) image analysis. After 8 weeks of being fed a high-fat diet, KK/HIJ mice were treated with 300 mg/kg/day of CS extract and 0.5 mg/kg/day of rosiglitazone (RS) for 4 consecutive weeks and were compared to the control group (HFD). The PPAR-*γ* protein expression levels were evaluated in liver (compared to *β*-actin) at the end of the test period. Data are expressed as the mean ± SE (*n* = 8 for each group); *P < 0.05, as compared to the control group.

In order to elucidate the insulin-sensitizing effects of RS and CS, we employed western blot analysis and found that mice treated with RS had dramatically increased levels of expression of PPAR-*γ* protein in liver tissues. However, the administration of CS did not effectively increase PPAR-*γ* protein levels over that of the control group (Figure 5).

### 3.6. Na^+^/K^+^-ATPase Activity

**Figure 6:**
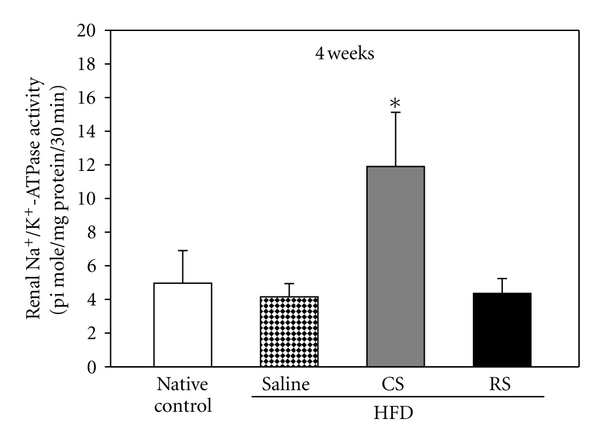
Renal Na^+^/K^+^-ATPase activity. Na^+^/K^+^-ATPase activity in the renal cortex of KK/HIJ mice (HFD) after treatment with 300 mg/kg/day of *Cordyceps sinensis* (CS) and 0.5 mg/kg/day of rosiglitazone (RS) for consecutive 4 weeks as compared to native controls (normal diet) and the control group (HFD). Data are expressed as mean ± SE (*n* = 8 for each group); *P < 0.05, as compared to the control group.

Enzyme activity assays revealed that Na^+^/K^+^-ATPase activity in the renal cortex was significantly elevated after 4 weeks of CS treatment compared to that of control and RS groups (Figure 6).

### 3.7. Kidney MT Staining

**Figure 7:**
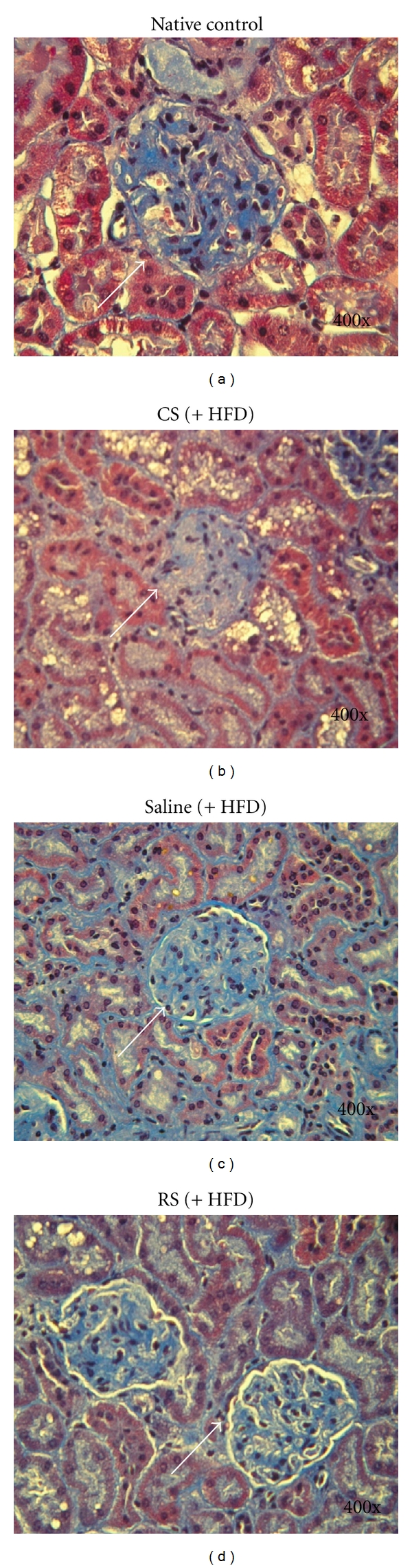
Histopathological examination of the renal cortex using Masson's trichrome staining. Histopathological study of the renal cortex in native KK/HIJ and HFD-fed KK/HIJ mice after treatment with either 300 mg/kg/day *Cordyceps sinensis* (CS), 0.5 mg/kg/day rosiglitazone (RS), or the control group (saline) for 4 consecutive weeks. (a) In the native control group, the glomerulus (white arrow) remained relatively intact with positive collagen staining; weak collagen staining was present in tubules and the interstitium. (b) In KK/HIJ (HFD) mice treated with saline, obvious mesangial matrix accumulation with diffuse collagen fibril deposition in different compartments was observed. (c) In KK/HIJ mice treated with CS, the matrix accumulation and collage staining was less severe than that in controls. (d) In KK/HIJ mice treated with RS, obvious mesangial matrix accumulation and diffuse collagen staining within renal tissues were observed (Masson trichrome, 400x).

We examined mouse renal cortical slices using Masson's trichrome (MT) stain to detect the severity of overt nephropathy as indicated by collagen fibril deposition in glomeruli, tubules, and the interstitium. As shown in Figure 7, the renal cortex of native KK/HIJ mice who were fed a normal diet showed positive collagen staining within glomeruli (which remain relatively intact) but weak collagen staining in the tubules and interstitium (Figure 7(a)). After 4 weeks of being fed a high-fat diet, glomerular mesangial matrix accumulation and strong collagen staining in glomeruli, tubules, and interstitium were apparent in tissues from the control group (HFD) (Figure 7(b)). In samples from mice treated with CS, the degree of mesangial matrix accumulation and collagen staining in different renal compartments seemed much improved over that of the control group (Figure 7(c)). However, samples from mice treated with RS showed no obvious improvement in collagen deposition and mesangial matrix accumulation compared to that of controls (Figure 7(d)).

## 4. Discussion

The hypoglycemic activity of CS in genetic and experimentally induced diabetic mouse models has been previously described [13, 14, 16, 17]. After daily oral, intravenous, or intraperitoneal administration of CS extract for several weeks, significant reductions in fasting blood glucose levels, attenuation of polydipsia, and improvements in glucose tolerance have been observed in several streptozotocin-induced and genetic diabetic animal models. Some CS extract products were also proven to increase insulin sensitivity [21, 22]. The main active ingredients of CS have been shown to be polysaccharides, major components of fungal structures. Despite this evidence, information regarding the influence of CS extracts on pancreatic *β* cells and renal tissue in type 2 DM animal models is still lacking. Our study demonstrated that ethanol extracts of CS had no obvious hypoglycemic activity and no ability to induce insulin sensitization. However, we found that CS confers significant protective effects in several tissues in our type 2 DM mouse model, potentially reducing DM-related complications such as diabetic nephropathy and *β*-cell destruction.

Similar to the findings of previous animal studies, our in vivo study showed increased blood insulin levels in these mice treated with CS, which may be due to the stimulation of pancreatic *β* cells [17, 22]. In addition, mice treated with CS had less body weight gain and better glucose tolerance than did controls, although these data were not statistically significance. Our results differ from those of previous studies in several ways. Initially, serum total cholesterol and triglyceride levels in our KK/HIJ diabetic mice had no obvious improvement after CS administration; but the HDL/LDL ratio increased after 4 weeks, implying that CS was capable of improving lipid profiles. On the other hand, insulin sensitivity did not improve significantly as indicated by the expression levels of hepatic PPAR-*γ* protein, which has been shown to correlate well with insulin sensitivity in several animal models [23–25]. One possible reason for the lack of effect could be that the ethanol extract from CS might contain several other non-polysaccharide components that could play an important role in inducing the previously observed hypoglycemic effects. In addition, the KK/HIJ mouse strain has potentially severe insulin resistance, which may make the degree of improvement difficult to evaluate while using lower doses of CS extract [26–28].

Interestingly, although no obvious improvement in insulin sensitivity was noted after CS extract use, in vitro cytoxicity assays demonstrated that pancreatic *β*-cell viability increased dramatically with CS extract treatment, implying that CS protects *β* cells. From a clinical perspective, preservation of *β*-cell function and survival should be taken into consideration when choosing medications for DM [29]. Sulfonylureas, which are often used as the first line of defense in diabetes therapy, had been found to accelerate *β*-cell exhaustion and apoptosis [30]. In some diabetic animal studies, certain drugs have demonstrated the ability to rescue or rejuvenate beta cells; such drugs include an ATP-sensitive K^+^ channel opener (diazoxide) [31], the thiazolidinediones (TZDs) [32], and newly developed incretin mimetics and enhancers [33]. Therefore, the potential ability of CS extract to preserve beta-cell function may afford a promising therapy for DM. This is an important finding from our in vitro studies.

Diabetic nephropathy is the leading cause of end-stage renal disease, affecting more than 40% of diabetic patients worldwide. It is also one of the most significant long-term complications in morbidity and mortality for diabetic patients [34]. Therefore, studies of the pathophysiology and mechanisms of diabetic nephropathy are very important. Renal Na^+^/K^+^ ATPase (NKA), a major transporter of renal sodium, may play a role in diabetic nephropathy. In one study of STZ-induced diabetes in rats, NKA activity was found to be decreased by 40% in erythrocytes, sciatic nerves, and kidneys [35]. NKA activity is also decreased in the medullary thick ascending limb in of STZ-induced diabetic rats 12 weeks after induction [36]. The activity of renal NKA was found to be downregulated in animal models of chronic hyperglycemia, and advanced glycation end products may play an important role in this process [37]. Due to the difficulty of accessing renal tissue in human studies, NKA activity has usually been measured via erythrocytes. Several studies have shown decreased erythrocyte NKA activity in diabetic patients [38, 39]. However, the effects on preservation of NKA are not observed while treated with RS. Therefore, decreased NKA activity is thought to be a potential risk factor for the development of diabetic nephropathy in both animals and humans, especially while the disease is in progression [40]. In our study, NKA activity significantly increased after the use of CS extract for several weeks. Whether the CS extract has a renoprotective effect still remains to be determined by further investigations.

Our study had several limitations. First, some of our results have a trend suggesting that CS extract leads to improvements in biochemical laboratory parameters, but without significance. More significant impacts might be “masked” due to severe insulin resistance in KK/HIJ mice. Despite this limitation, no previous studies have investigated the glucose-lowering effects of solid-state fermented Cordyceps sinensis to this extent. Second, the duration of CS extract administration may be short (4 weeks). Nevertheless, the effects of CS on pancreatic beta cell preservation, increased renal NKA activity, and reduced renal collagen deposition are still significant.

In conclusion, this preliminary study suggests that CS extract promotes *β*-cell survival, increases renal NKA activity, and decreases collagen deposition and mesangial matrix accumulation. These beneficial actions indicate that CS might be a potential drug candidate for retardation of pancreatic *β*-cell failure and diabetic nephropathy. In future studies, we plan to use different diabetic mouse models with less insulin resistance than KK/HIJ mice and increase the duration of CS extract administration.

##  Conflict of Interests

The authors declare that they have no conflict of interests.

##  Authors' Contribution

W.-C. Kan and H.-Y. Wang contributed equally to this work.

## References

[1] Winer N, Sowers JR (2004). Epidemiology of diabetes. *Journal of Clinical Pharmacology*.

[2] Deshpande AD, Harris-Hayes M, Schootman M (2008). Epidemiology of diabetes and diabetes-related complications. *Physical Therapy*.

[3] Reaven GM (2005). Insulin resistance, the insulin resistance syndrome, and cardiovascular disease. *Panminerva Medica*.

[4] Bloomgarden ZT (2004). Definitions of the insulin resistance syndrome: the 1st World Congress on the Insulin Resistance Syndrome. *Diabetes Care*.

[5] Chen D, Wang MW (2005). Development and application of rodent models for type 2 diabetes. *Diabetes, Obesity and Metabolism*.

[6] Islam MS, Loots DT (2009). Experimental rodent models of type 2 diabetes: a review. *Methods and Findings in Experimental and Clinical Pharmacology*.

[7] Srinivasan K, Ramarao P (2007). Animal models in type 2 diabetes research: an overview. *Indian Journal of Medical Research*.

[8] Accili D, Kido Y, Nakae J, Lauro D, Park BC (2001). Genetics of type 2 diabetes: insight from targeted mouse mutants. *Current Molecular Medicine*.

[9] Karasawa H, Nagata-Goto S, Takaishi K, Kumagae Y (2009). A novel model of type 2 diabetes mellitus based on obesity induced by high-fat diet in BDF1 mice. *Metabolism*.

[10] Terauchi Y, Kadowaki T (2002). Insights into molecular pathogenesis of type 2 diabetes from knockout mouse models. *Endocrine Journal*.

[11] Paterson RRM (2008). *Cordyceps*: a traditional Chinese medicine and another fungal therapeutic biofactory?. *Phytochemistry*.

[12] Zhou X, Gong Z, Su Y, Lin J, Tang K (2009). *Cordyceps* fungi: natural products, pharmacological functions and developmental products. *Journal of Pharmacy and Pharmacology*.

[13] Kiho T, Hui J, Yamane A, Ukai S (1993). Polysaccharides in Fungi. XXXII. Hypoglycemic activity and chemical properties of a polysaccharide from the cultural mycelium of *Cordyceps sinensis*. *Biological and Pharmaceutical Bulletin*.

[14] Kiho T, Ookubo K, Usui S, Ukai S, Hirano K (1999). Structural features and hypoglycemic activity of a polysaccharide (CS-F10) from the cultured mycelium of *Cordyceps sinensis*. *Biological and Pharmaceutical Bulletin*.

[15] Guo JY, Han CC, Liu YM (2010). A contemporary treatment approach to both diabetes and depression by *Cordyceps sinensis*, Rich in Vanadium. *Evidence-Based Complementary and Alternative Medicine*.

[16] Lo HC, Tu ST, Lin KC, Lin SC (2004). The anti-hyperglycemic activity of the fruiting body of *Cordyceps* in diabetic rats induced by nicotinamide and streptozotocin. *Life Sciences*.

[17] Lo HC, Hsu TH, Tu ST, Lin KC (2006). Anti-hyperglycemic activity of natural and fermented *Cordyceps sinensis* in rats with diabetes induced by nicotinamide and streptozotocin. *American Journal of Chinese Medicine*.

[18] Zhang C, Zou X, Leluo G, Xu J, Xiang M (2008). Prevention of type 1 diabetes by immature dendritic cells treated with an ethanol extract of *Paecilomyces hepiali* Chen mycelium. *Methods and Findings in Experimental and Clinical Pharmacology*.

[19] Zhang Z, Wang X, Zhang Y, Ye G (2011). Effect of *Cordyceps sinensis* on renal function of patients with chronic allograft nephropathy. *Urologia Internationalis*.

[20] Tang R, Zhou Q, Shu J (2009). Effect of *Cordyceps sinensis* extract on Klotho expression and apoptosis in renal tubular epithelial cells induced by angiotensin II. *Journal of Central South University*.

[21] Balon TW, Jasman AP, Zhu JS (2002). A fermentation product of *Cordyceps sinensis* increases whole-body insulin sensitivity in rats. *Journal of Alternative and Complementary Medicine*.

[22] Zhang X, Liu YK, Zheng Q, Shen W, Shen DM (2003). Influence of *Cordyceps sinensis* on pancreatic islet beta cells in rats with experimental liver fibrogenesis. *Zhonghua Gan Zang Bing Za Zhi*.

[23] Hsiao G, Chapman J, Ofrecio JM (2011). Multi-tissue, selective PPAR*γ* modulation of insulin sensitivity and metabolic pathways in obese rats. *American Journal of Physiology*.

[24] Balasubramanian R, Gerrard J, Dalla Man C (2010). Combination peroxisome proliferator-activated receptor *γ* and *α* agonist treatment in Type 2 diabetes prevents the beneficial pioglitazone effect on liver fat content. *Diabetic Medicine*.

[25] Mollah ML, Kim GS, Moon HK (2009). Antiobesity effects of wild *ginseng* (Panax ginseng C.A. meyer) mediated by PPAR-*γ*, GLUT4 and LPL in ob/ob mice. *Phytotherapy Research*.

[26] Svenson KL, Von Smith R, Magnani PA (2007). Multiple trait measurements in 43 inbred mouse strains capture the phenotypic diversity characteristic of human populations. *Journal of Applied Physiology*.

[27] Polotsky VY (2007). Mouse model of the metabolic syndrome: the quest continues. *Journal of Applied Physiology*.

[28] Qi Z, Fujita H, Jin J (2005). Characterization of susceptibility of inbred mouse strains to diabetic nephropathy. *Diabetes*.

[29] Wajchenberg BL (2007). *β*-cell failure in diabetes and preservation by clinical treatment. *Endocrine Reviews*.

[30] Del Prato S, Pulizzi N (2006). The place of sulfonylureas in the therapy for type 2 diabetes mellitus. *Metabolism*.

[31] Matsuda M, Kawasaki F, Mikami Y (2002). Rescue of beta-cell exhaustion by diazoxide after the development of diabetes mellitus in rats with streptozotocin-induced diabetes. *European Journal of Pharmacology*.

[32] Walter H, Lübben G (2005). Potential role of oral thiazolidinedione therapy in preserving *β*-cell function in type 2 diabetes mellitus. *Drugs*.

[33] Wajchenberg BL (2010). Clinical approaches to preserve beta-cell function in diabetes. *Advances in Experimental Medicine and Biology*.

[34] http://www.usrds.org.

[35] Raccah D, Lamotte-Jannot MF, Issautier T, Vague P (1994). Effect of experimental diabetes on Na/K-ATPase activity in red blood cells, peripheral nerve and kidney. *Diabete et Metabolisme*.

[36] Tsimarato M, Coste TC, Djemli-Shipkolye A (2001). Evidence of time-dependent changes in renal medullary Na,K-ATPase activity and expression in diabetic rats. *Cellular and Molecular Biology*.

[37] Gallicchio MA, Bach LA (2010). Advanced glycation end products inhibit Na^+^ K^+^ ATPase in proximal tubule epithelial cells: role of cytosolic phospholipase A2*α* and phosphatidylinositol 4-phosphate 5-kinase *γ*. *Biochimica et Biophysica Acta*.

[38] Jannot MF, Raccah D, De La Tour DD, Coste T, Vague P (2002). Genetic and environmental regulation of Na/K adenosine triphosphatase activity in diabetic patients. *Metabolism*.

[39] Vague P, Coste TC, Jannot MF, Raccah JD, Tsimaratos M (2004). C-peptide, Na^+^,K^+^-ATPase, and diabetes. *Experimental Diabesity Research*.

[40] Shahid SM, Mahboob T (2008). Electrolytes and Na^+^-K^+^-ATPase: potential risk factors for the development of diabetic nephropathy. *Pakistan Journal of Pharmaceutical Sciences*.

